# Antibiotic Resistance Trends in Carbapenem-Resistant Gram-Negative Pathogens and Eight-Year Surveillance of XDR Bloodstream Infections in a Western Greece Tertiary Hospital

**DOI:** 10.3390/pathogens13121136

**Published:** 2024-12-23

**Authors:** Maria Lagadinou, Marina Amerali, Christos Michailides, Anna Chondroleou, Katerina Skintzi, Anastasia Spiliopoulou, Fevronia Kolonitsiou, Leonidia Leonidou, Stelios F. Assimakopoulos, Markos Marangos

**Affiliations:** 1 Department of Internal Medicine, University Hospital of Patras, 265 04 Patras, Greeceamerali.marina@gmail.com (M.A.); christos.mich1@gmail.com (C.M.); lydleon@yahoo.com (L.L.); mmarangos@yahoo.com (M.M.); 2 Department of Microbiology, Medical School, University of Patras, 265 04 Patras, Greece; spil@upatras.gr (A.S.); kolonits@upatras.gr (F.K.); 3 Nurse Infection Control, University Hospital of Patras, 265 04 Patras, Greece; anna_chondroleos@yahoo.gr (A.C.); katrinski80@gmail.com (K.S.); 4 Department of Microbiology, University Hospital of Patras, 265 04 Patras, Greece; 5 Division of Infectious Diseases, University Hospital of Patras, 265 04 Patras, Greece

**Keywords:** extensive drug resistance, bacteria, antibiotics, surveillance

## Abstract

**Background**: The increased prevalence of antibiotic resistance among Gram-negative bacteria presents a severe public health challenge, leading to increased mortality rates, prolonged hospital stays, and higher medical costs. In Greece, the issue of multidrug-resistant Gram-negative bacteria is particularly alarming, exacerbated by overuse of antibiotics and inadequate infection control measures. This study aimed to detect the prevalence of extensively drug-resistant (XDR) Gram-negative bacteria in a tertiary hospital in Western Greece over the last eight years from 2016 to 2023. **Materials and Methods**: In the present study, all Carbapenem-resistant (CR) *Acinetobacter baumannii*, *K. pneumoniae* and *Pseudomonas aeruginosa*. bloodstream isolates from patients hospitalized in the University General Hospital of Patras in Western Greece, from January 2016 to December 2023, were recorded. XDR strains were defined as non-susceptible to at least one agent in all but two or fewer antimicrobial categories (i.e., bacterial isolates remain susceptible to only one or two categories). The prevalence and distribution of these pathogens across different hospital wards and their susceptibility patterns to key antibiotics (aminoglycosides, trimethoprim-sulfamethoxazole, tigecycline, colistin, ampicillin-sulbactam, ceftolozane-tazobactam and ceftazidime-avibactam) were recorded. Results: A total of 1142 blood cultures growing carbapenem-resistant *Klebsiella pneumoniae* (CRKp), *Acinetobacter baumannii* (CRAB) and *Pseudomonas aeruginosa* (CRPsA) were studied. In the present study, we found an increased resistance of both *A. baumannii* and *K. pneumoniae* in colistin. *Acinetobacter baumannii* had colistin resistance rates between 8.4% and 49.3%, showing a stable increase during the study period. *K. pneumoniae* showed an increased colistin-resistance rate in 2022 and 2023 (46.8% and 31.2%, respectively) Regarding *P. aeruginosa,* amikacin was almost inactive with a rate 68.4% and 87.5% in 2020 and 2023, respectively. Of all CR isolates, 69.3% were extensively drug-resistant (XDR). *Acinetobacter baumannii* had the highest percentage of XDR isolates (34.3%), followed by *K. pneumoniae* (26.8%) and *P. aeruginosa* (8.1%). Most XDR pathogens were isolated from the ICU (73.4%), followed by the internal medicine units (64%) and surgical units (22%). **Conclusions**: The rate of antimicrobial resistance and extensive drug resistance is alarmingly high, which calls for strict surveillance, control measures, and antibiotic stewardship to prevent the development of further resistance.

## 1. Introduction

Antimicrobial resistance has been recognized as a great global public health issue [1]. The rising incidence of resistance among Gram-negative bacteria poses a serious threat to the management of nosocomial infections [2]. Various mechanisms leading to Gram-negative bacterial resistance have been identified. Based on the patterns of antibiotic resistance, bacteria can be classified as multidrug-resistant (MDR), extensively drug-resistant (XDR), or pan drug-resistant (PDR). The Center for Disease Control & Prevention (CDC) and the European Centre for Disease Control (ECDC) have developed standardized definitions of all these entities, which are now universally accepted [3].

The emergence and spread of extensively drug-resistant (XDR) Gram-negative bacteria, such as *Klebsiella pneumoniae*, *Pseudomonas aeruginosa*, and *Acinetobacter baumannii*, presents a critical challenge for healthcare systems worldwide. In Southern Europe, particularly in Italy and Greece, the epidemiology of these pathogens has shown a concerning rise in recent years. These XDR strains are associated with high morbidity and mortality, significantly affecting patient outcomes and escalating healthcare costs [4]. Moreover, bloodstream infections caused by carbapenem-resistant bacteria, especially bacteremia caused by XDR strains with limited therapeutic options, are linked to significantly high mortality rates, presenting a serious threat to patient survival and healthcare systems [5]. Even more, Petersiel et al. highlighted that nosocomial bloodstream infections (BSIs), especially those caused by multidrug-resistant bacteria, are associated with significantly high mortality rates. Additionally, among patients who survive hospitalization, these infections are linked to a decline in functional status [6]. Among hospital survivors, BSIs are associated with functional decline. Murray C.J.L. et al. reported that in 2019, bacterial antimicrobial resistance (AMR) was associated with an estimated 4.95 million deaths (3.62–6.57 million), including 1.27 million deaths (95% UI 0.911–1.71 million) directly attributable to AMR. Regionally, the highest all-age death rate attributable to AMR was observed in western sub-Saharan Africa, with 27.3 deaths per 100,000 (20.9–35.3), while the lowest was in Australasia, at 6.5 deaths per 100,000 (4.3–9.4) [7].

A recent study in Greece by Karakosta et al. highlighted a notable rise in Carbapenem-resistant (CR) Gram-negative bacteria bloodstream infections (BSIs) caused by multidrug-resistant organisms (MDROs) across three tertiary hospitals between 2019 (pre-COVID-19) and 2022 (during the COVID-19 pandemic) [8]. Additionally, in both Italy and Greece, the prevalence of XDR Gram-negative bacteria has increased dramatically, driven by factors such as the overuse of antibiotics and inadequate infection control measures [7]. According to the ECDC resistance rates have escalated, with Italy and Greece reporting some of the highest incidences of carbapenem-resistant *Enterobacteriaceae* and *Acinetobacter* spp. [9]. These pathogens contribute significantly to the burden of nosocomial infections, leading to prolonged hospital stays, increased treatment costs, and elevated mortality rates [4]. Furthermore, bloodstream infections caused by Carbapenem-resistant *Enterobacterales* (CREs), particularly those involving XDR strains with limited therapeutic options, are associated with significantly high mortality rates, posing a critical threat to patient survival and healthcare systems [5].

Our study aims to provide a detailed epidemiological analysis of XDR Gram-negative bacteria in a tertiary university hospital in Western Greece from 2016 to 2023. We will focus on the distribution of these pathogens across different hospital wards and their susceptibility patterns to key antibiotics. Understanding these patterns is crucial for developing effective antibiotic stewardship and infection control strategies and improving patient outcomes.

## 2. Materials and Methods

### 2.1. Study Design

In this study, all bloodstream isolates of carbapenem-resistant *Acinetobacter baumannii*, *Klebsiella pneumoniae*, and *Pseudomonas aeruginosa* from patients hospitalized at the University General Hospital of Patras in Western Greece, were documented. The data collection spanned from January 2016 to December 2023. Patient records were obtained from four key departments: Medical Wards (MW), which included Internal Medicine, Cardiology, Nephrology, Neurology, Hematology-Oncology, and the Hematopoietic Stem Cell Transplantation Unit; Surgical Departments (SD), covering General Surgery, Orthopedics, Obstetrics, Neurosurgery, and Urology; the adult Intensive Care Unit (ICU); and both the Neonatal Intensive Care Unit (NICU) and the Pediatric Intensive Care Unit (PICU). Extensively drug-resistant bacteria were classified based on the criteria established by the CDC [5]. The isolation of pathogens was evaluated in terms of the presence of fever.

The study was carried out in accordance with the Hospital Research Ethics Committee’s guidelines (Approval Number PN: 10408), the Declaration of Helsinki, and STROBE guidelines.

### 2.2. XDR Definition

According to the definitions given in 2008, XDR is defined as non-susceptible to at least one agent in all but two or fewer antimicrobial categories meaning the bacterial isolates remain susceptible to only one or two categories [5]. Regarding *Acinetobacter*, due to the lack of established clinical breakpoints for tigecycline by EUCAST, strains with a tigecycline minimum inhibitory concentration (MIC) greater than 2 mg/mL were classified as resistant [8]. Previous clinical studies with XDR *A. baumannii* infections have shown that when tigecycline MIC is >2 μg/mL, significantly higher therapeutic failures and mortality were observed either when tigecycline was used as monotherapy or as part of a combination therapy with colistin [10,11,12].

### 2.3. Bacterial Identification and Antimicrobial Susceptibility Testing

The identification of Gram-negative bacteria was carried out using VITEK^®^ 2 Gram-negative identification cards (bioMérieux, Marcy-l’Etoile, France). The study evaluated susceptibility to various antimicrobial agents, including amikacin (AMK), tigecycline (TGC), colistin (CS), trimethoprim-sulfamethoxazole (SXT), ceftolozane-tazobactam (C/T), ampicillin-sulbactam (SAM), and ceftazidime-avibactam (CZA). In selected cases, other methods were used for antimicrobial susceptibility testing such as disks or antibiotic gradient strips.

The selection of these antimicrobial agents was guided by the European Committee for Antimicrobial Susceptibility Testing (EUCAST) guidelines, as well as recommendations from the ECDC and the CDC [9]. The results were assessed according to EUCAST guidelines [EUCAST Clinical Breakpoint Tables version 14.0, valid from 1 January 2024], with isolates categorized as susceptible (including susceptible, increased exposure) or resistant [8]. Colistin’s MIC was determined using the broth microdilution method (Sensi Test TM Colistin, Liofilchem, Roseto degli Abruzzi, Italy), as recommended. Susceptibility to tigecycline was defined as an MIC of less than 2 μg/mL [13,14,15].

### 2.4. Statistical Analyses

Data were statistically analyzed using Statistical Package for Social Sciences (SPSS) version 22.0 for Windows (IBM Corp., Armonk, NY, USA). Frequencies and percentages of XDR bacteria were determined for all clinical isolates. The chi-square test was used to determine the statistical difference between 2016, which was the first year that data collection began, and the year in which the greatest variation was observed for the two pathogens with the highest prevalence.

## 3. Results

### 3.1. XDR A. baumanii, K. pneumoniae, and P. aeruginosa Isolation

During the study period, a total of 1142 blood cultures of carbapenem-resistant *Klebsiella pneumoniae* (CRKp), *Acinetobacter baumannii* (CRAB), and *Pseudomonas aeruginosa* (CRPsA) were recorded. Among these carbapenem-resistant Gram-negative pathogens isolated from bloodstream infections, 791 cases (69.3%) were due to extensively drug-resistant (XDR) pathogens. Specifically, XDR *Acinetobacter baumannii* was identified in 392 cases, accounting for 34.3% of BSIs; XDR *K. pneumoniae* was found in 306 cases (26.8%), and XDR *P. aeruginosa* was detected in 93 cases (8.1%). All above-mentioned findings are presented in Table 1.

**Figure 1:**
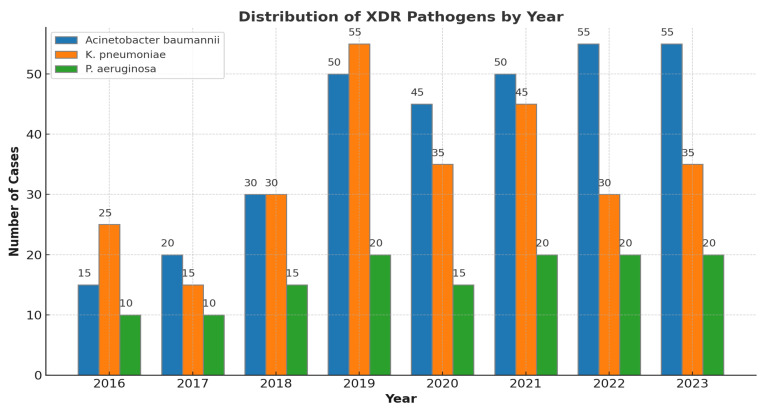
XDR *A*. *baumannii*, *K. pneumoniae*, and *P. aeruginosa* bloodstream infections per year.

Most cases occurred in Intensive Care Units (ICUs) (74.3%), followed by medical wards (64%) and surgical wards (22%) (Table 2). The yearly distribution of XDR isolates is presented in Figure 1. Notably, our data indicate a statistically significant increase in the prevalence of XDR *Acinetobacter baumannii* after 2019 (15% in 2016 vs. 50% in 2019, *p*: 0.017), while the prevalence of XDR *K. pneumoniae* decreased during the same period (55% in 2019 vs. 30% in 2022, *p*: 0035), except for a peak in 2021.

### 3.2. Department Distribution of XDR A. baumanii, K. pneumoniae, and P. aeruginosa Bloodstream Infections Per Year

**Figure 2:**
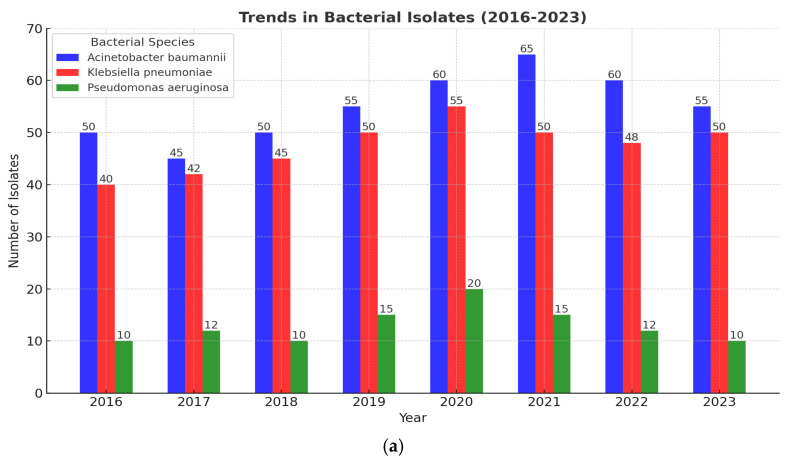

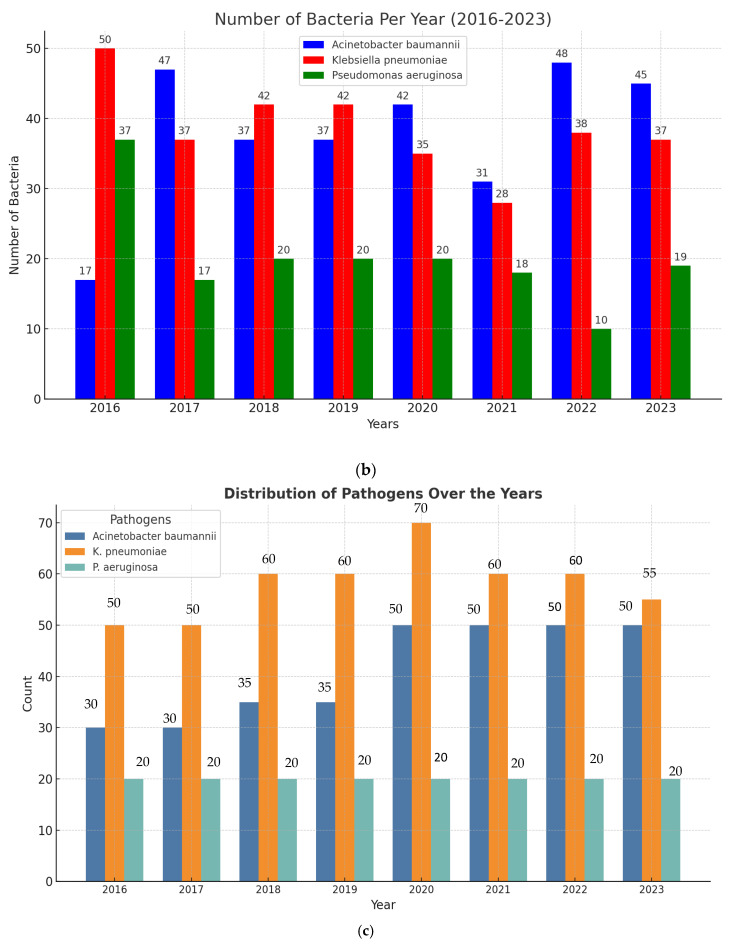
(**a**) Distribution of XDR pathogens in Intensive Care Units (ICU) (neonatal and adult). (**b**) Distribution of XDR pathogens in medical wards including Internal Medicine, Cardiology, Nephrology, Neurology, Hematology–Oncology, and Hematopoietic Stem Cell Transplantation Unit. (**c**) Distribution of XDR pathogens in Surgical wards.

The department distribution of XDR Gram-negative BSIs is presented in Figure 2a–c. As shown, XDR *A. baumannii* BSIs became the most prevalent after 2020, except in 2021, when XDR K. pneumoniae predominated in the surgical wards.

### 3.3. Antimicrobial Resistance Rates of Carbapenem-Resistant A. *baumannii*, K. pneumoniae and P. aeruginosa BSI Isolates

**Figure 3:**
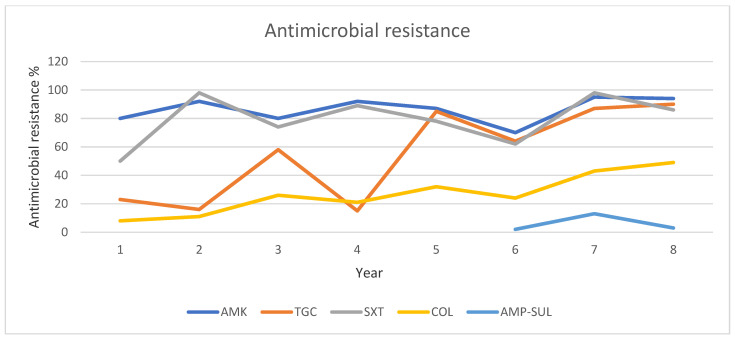
Trends of antibiotic resistance of CR *A. baumannii*, isolated in BSIs during 2016–2023. Abbreviations: AMK: Amikacin, AMP/SULB: ampicillin-sulbactame, TGC: Tigecycline, SXT: Trimethoprime-sulfamethoxazole, COL: colistin.

**Figure 4:**
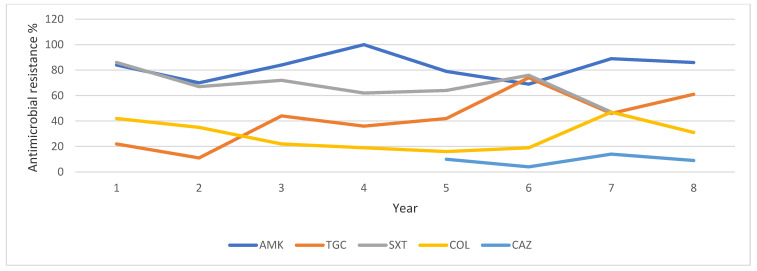
Trends of antibiotic resistance of CR *K. pneumoniae* isolated in BSIs during 2016–2023. Abbreviations: AMK: Amikacin, TGC: Tigecycline, SXT: Trimethoprime-sulfamethoxazole, COL: colistin, CAZ: ceftazidime-avibactam.

**Figure 5:**
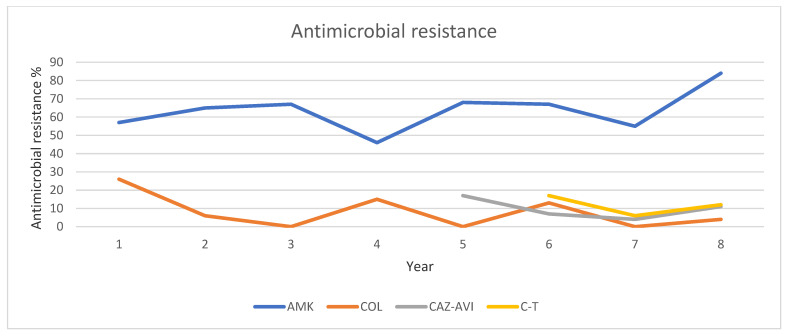
Trends of antibiotic resistance of CR *P. aeruginosa* isolated in BSIs during 2016–2023.Abbreviations: AMK: amikacin, COL: colistin, CAZ-AVI: ceftazidime-avibactam, C-T: ceftolozane-tazobactam.

The resistance rates for the entire CREs are presented in Table 3, Table 4 and Table 5 and Figure 3, Figure 4 and Figure 5. Regarding *Acinetobacter baumannii* colistin-resistance rate ranged from 8.4% to 49.3% showing a stable increase during the study period. The resistance rate to trimethoprim/sulfamethoxazole was between 50% and 98.2%. Tigecycline demonstrated resistance rates of 87% and 89.6% in 2022 and 2023, respectively. It is noteworthy that very high intermediate susceptibility rates of *Acinetobacter baumannii* to ampicillin/sulbactam in 2021, 2022, and 2023 were demonstrated (Table 3, Figure 3).

Regarding *K. pneumoniae*, the ceftazidime-avibactam resistance rate was 10% in 2020 and reached up to 13.6% in 2022. Remarkably, colistin resistance rates were low, except for the years 2016 and 2022 (Table 4, Figure 4).

Concerning *P. aeruginosa,* the ceftazidime-avibactam resistance rate was 17.2% in 2020 showing a steady decrease throughout the study period. Ceftolozane-tazobactam resistance rate was 17% in 2021, 6% in 2022, and 12% in 2023 (Table 5, Figure 5).

## 4. Discussion

Healthcare-associated infections (HAIs) remain a major global concern, particularly due to the increasing prevalence of MDR and XDR bacteria [16]. Previous studies have reported that for 100 patients admitted to the hospital, 7 patients in high-income countries and 10 patients in developing and low-income economies had at least one type of HAI [17]. In 2016, the European Centre for Disease Prevention and Control (ECDC) estimated that the burden of six main types of healthcare-associated infection (healthcare-associated pneumonia, urinary tract infection, surgical site infection, Clostridium difficile infection, neonatal sepsis, and primary bloodstream infection) expressed in disability-adjusted life years (DALYs) in the European Union and European Economic Area (EU/EEA) was higher than the combined burden of 31 other infectious diseases under surveillance by the ECDC [18]. The present study examines the detection rates and resistance patterns of bacteria isolated at a tertiary university hospital in Western Greece from 2016 to 2023, with a specific focus on XDR pathogens.

Our findings reveal a gradual increase in the detection rates of XDR pathogens, particularly following the COVID-19 pandemic, consistent with global trends. Similarly, de Kraker, M. E., et al. reported that the burden of bacteremias is increasing in European hospitals. Notably, the expansion of antimicrobial resistance seems to create an additional strain on healthcare systems by further increasing the burden of disease caused by difficult-to-treat bacteremias in Europe [19]. On the other hand, Kadri S et al. reported that between 2009 and 2013, 471 (1%) of 45,011 Gram-negative BSI episodes at 92 (53.2%) of 173 hospitals exhibited difficult-to-treat resistance, ranging from 0.04% for *Escherichia coli* to 18.4% for *Acinetobacter baumannii* [18]. Notably, in the present study, 69.3% of Gram-negative bacteria isolated from blood cultures were identified as XDR, highlighting a significant public health challenge. This percentage is higher than that reported in similar studies, indicating a growing resistance among Gram-negative pathogens in our region [3].

*A. baumannii* emerged as the most prevalent resistant pathogen in our study, with 34.3% of its isolates being XDR. This finding is consistent with recent data from the European Antimicrobial Resistance Surveillance Network (EARS-Net), which reported a significant increase in *Acinetobacter* spp. bloodstream infections (BSIs) in the European Union and European Economic Area during the COVID-19 pandemic. Our data showed a steady rise in resistance, particularly after 2019, highlighting the ongoing threat posed by this pathogen [20,21].

*K. pneumoniae* is recognized as the second most common Gram-negative microorganism causing bloodstream infections in both community and nosocomial settings [22]. In our study, XDR *K. pneumoniae* cases accounted for 26.8%. In contrast, a large study conducted in Canada involving 640 episodes of *K. pneumoniae* bacteremia reported an overall annual population incidence of 7.1 per 100,000 [17]. Interestingly, while other studies have reported high frequencies of XDR *K. pneumoniae*, our data indicated a decrease in its isolation rates from 2021 to 2023 [17]. This reduction can be attributed to the introduction of new antibiotics, such as ceftazidime-avibactam, which have proven effective against carbapenem-resistant *K. pneumoniae* strains. A study by Tumbarello et al. demonstrated significantly lower 30-day mortality rates in patients with bacteremic KPC-Kp infections treated with ceftazidime-avibactam. Effective treatment of XDR *K. pneumoniae* strains permits microbiological eradication, reducing the time of pathogen isolation by infected patients and consequently the risk of hospital transmission [23].

*P. aeruginosa* presented lower rates of extended drug resistance (8.1%) in our study. This contrasts with other studies reporting higher XDR rates [1,24]. For instance, a multicenter study reported 35.8% of *P. aeruginosa* isolates XDR, highlighting the variability in resistance patterns across different regions and healthcare settings [1].

Infections caused by XDR Gram-negative pathogens pose a significant therapeutic challenge both in our hospitals and across other healthcare facilities in the wider Mediterranean region. Over the past decade, antimicrobial resistance among *Acinetobacter baumannii* has increased considerably. Ahmad S. et al. reported that, among 114 *Acinetobacter* spp. infections, 96.5% were resistant to one or more drugs [25]. In the same study, tigecycline resistance rates reached 21.1%, while 96.49% of strains remained susceptible to colistin. Regarding *Klebsiella pneumoniae*, a recent study by Sekowska et al. found that, among pan-drug-resistant *K. pneumoniae* isolates, colistin exhibited the highest susceptibility rate at 43.9% [19]. Ceftazidime–avibactam demonstrated good activity against *K. pneumoniae* strains producing ESBLs, KPC, and OXA enzymes [26]. Additionally, a randomized clinical trial on abdominal infections reported that 98.6% of *Pseudomonas aeruginosa* isolates were susceptible to ceftolozane–tazobactam [27].

The distribution of these XDR pathogens varied significantly among different hospital wards. The highest percentage of XDR Gram-negative pathogens was identified in the ICU (74.3%), consistent with the well-established notion that ICUs are hotspots for antibiotic-resistant bacteria due to factors such as prolonged hospital stays, immunosuppression, and invasive procedures [28].

In the surgical wards, *Acinetobacter baumannii* accounted for a substantial proportion of XDR infections, correlating with the high usage of invasive devices and postoperative complications in these settings. The internal medicine wards also showed significant levels of XDR *K. pneumoniae*, potentially attributable to the high patient turnover and the complex comorbidities treated in these units.

Our study is the first to detail the epidemiology of XDR pathogens in a Greek tertiary hospital. The significant presence of XDR bacteria in bloodstream infections (BSIs) emphasizes the urgent need for enhanced infection control measures and judicious antibiotic use. The findings highlight the need for reinforced preventive strategies and effective treatment protocols tailored to local epidemiology. Moreover, this information guides public health interventions, including vaccination campaigns, awareness programs, or outbreak response efforts and helps clinicians anticipate the likelihood of encountering XDR pathogens in their practice.

Limitations of our study include the retrospective nature of the data analysis. Additionally, the unavailability of MIC data for new agents limited our ability to categorize microorganisms strictly according to standard definitions. Finally, our results are based on the evaluation of antimicrobial susceptibility phenotype and were not collated to the presence of resistance genes.

## 5. Conclusions

Our study highlights the alarming rate of XDR Gram-negative pathogens in nosocomial BSIs, emphasizing the critical need for improved infection control practices and personalized treatment approaches that consider local resistance patterns. Effective management of these infections remains a significant challenge, requiring a comprehensive strategy that includes both preventive and therapeutic measures.

## Figures and Tables

**Table 1:** Number of XDR cases among blood cultures of Carbapenem-resistant *Enterobacterales*.

Pathogen	Number of Cases	% of Total BSIs
Total XDR Pathogens	791	69.3%
XDR *A. baumannii*	392	34.3%
XDR *K. pneumoniae*	306	26.8%
XDR *P. aeruginosa*	93	8.1%

**Table 2:** Distribution of XDR pathogens.

Pathogen	ICUs	Medical Wards	Surgical Wards
XDR *A. baumanii*	176	120	96
XDR *K. pneumoniae*	120	110	76
XDR *P. aeruginosa*	45	30	18

**Table 3:** Antimicrobial resistance (%) of CR *A. baumannii* isolated in BSIs during 2016–2023.

	AMP/SULB	Amikacin	TGC	SXT	COL
2016	N/A	80	22.5	50	8.4
2017	N/A	91.3	16	98.2	10.5
2018	N/A	80	57.9	73.7	26.3
2019	N/A	91.5	15	89.4	21.3
2020	N/A	87	85	78.3	32.4
2021	2 ^1^	68.8	63.5	61.7	24.1
2022	12.5 ^2^	94.7	87	98.2	42.5
2023	2.9 ^3^	93.5	89.6	85.7	49.3

^1^: 98% showed intermediate susceptibility, ^2^: 87.5% showed intermediate susceptibility, ^3^: 97.1% showed intermediate susceptibility. Abbreviations: AMP/SULB: ampicillin-sulbactame, TGC: Tigecycline, SXT: Trimethoprime-sulfamethoxazole, COL: colistin.

**Table 4:** Antimicrobial resistance (%) of CR *K. pneumoniae* isolated in BSIs during 2016–2023.

	Amikacin	TGC	SXT	COL	CAZ-AVI
2016	83.7	22	85.5	42	
2017	70.4	11	66.7	35.2	
2018	84.2	43.5	71.7	21.7	-----
2019	100	36.2	61.7	19	
2020	79	42.3	63.6	16	10 ^1^
2021	68.5	74.1	75.9	18.5	3.7 ^2^
2022	89.2	46	74	46.8	13.6 ^3^
2023	85.9	61	76	31.2	8.7 ^4^

^1^: 0% MBL strains, ^2^: 0% MBL strains, ^3^: 2.5% MBL strains, ^4^: 0% MBL strains. Abbreviations: TGC: Tigecycline, SXT: Trimethoprime-sulfamethoxazole, COL: colistin. CAZ-AVI: ceftazidime-avibactam.

**Table 5:** Antimicrobial resistance (%) of CR *P. aeruginosa* isolated in BSIs during 2016–2023.

	Amikacin	COL	CAZ	C-T
2016	56.5	26	N/A	N/A
2017	65.1	6	N/A	N/A
2018	67	0	N/A	N/A
2019	46	14.5	N/A	N/A
2020	68.4	0	17.2 ^1^	N/A
2021	66.7	13.3	6.7 ^2^	17
2022	55	0	3.7 ^3^	6
2023	84	4.2	10.5 ^4^	12

^1^: 17.8% MBL strains, ^2^: 7.1% MBL strains, ^3^: 0% MBL strains, ^4^: 0% MBL strains. Abbreviations: COL: colistin, CAZ: ceftazidime-avibactam, C-T: ceftolozane-tazobactam.

## Data Availability

The datasets generated and analyzed during the current study are available upon reasonable request to the corresponding author.
